# Bidirectional Assessment of Stress, job satisfaction and work ability of Educators in day care centres: a real-time observation study - the study protocol (BASE)

**DOI:** 10.1186/1745-6673-5-16

**Published:** 2010-06-29

**Authors:** Bianca Kusma, Albert Nienhaus, Michael Spallek, David Quarcoo, David A Groneberg, Stefanie Mache

**Affiliations:** 1 Institute of Occupational Medicine, Charité - Universitätsmedizin Berlin, Free University and Humboldt University, Thielallee 69-73, 14195 Berlin, Germany; 2 Department of Respiratory Medicine, Hannover Medical School, Carl-Neuberg-Straße 1, 30625 Hannover, Germany; 3 Institution for Statutory Accident Insurance in the Health and Welfare Services, Pappelallee 35/37, 22089 Hamburg, Germany; 4 European Research Group on Environment and Health in the Transport Sector (EUGT e.V.), Thielallee 69, 14195 Berlin, Germany; 5 Department of Medicine/Psychosomatics, Charité - Universitätsmedizin Berlin, Free University and Humboldt University, Luisenstraße 13a, 10117 Berlin, Germany

## Abstract

**Background:**

Occupational demands of educators are not very well researched. Nevertheless their work is subject to several requirements. Whether these demands have an effect on the work ability and the health status of employees has also not been examined. Furthermore it is unclear if the ownership type of day care centres have an influence on job satisfaction and work ability of the pedagogical staff and what kind of resources do exist. Previous studies were mainly based on questionnaire data. Objective data does not exist. Therefore the aim of this investigation is to collect precise data relating to work of educators.

**Methods:**

Effects of different types of ownership of day care centres on job satisfaction and work ability of educators will be assessed with the help of objective real time studies in combination with multi-level psycho diagnostic measurements.

**Discussion:**

The present study is the first of its kind. Up to now there are no computer-based real time studies on workflow of pedagogical staff with regard to assess their work-related stress. Following an exhaustive documentation of educators work processes the day-to-day task can be estimated and approaches for prevention can be developed. This can substantially contribute to an overall improvement of child care in Germany.

## Background

### The current work situation of educators in German day care centres

Working conditions of educators have not been scrutinized thoroughly in scientific research. Only a few studies have so far examined workload and strain in this profession. It is subject to several psychosocial requirements [1]. These are to a lesser extent special severe burdens but rather an interaction of minor strains which sum up in their negative effect [2]. Among them are disadvantageous postures caused by inappropriate sized furniture, overexertion of the voice and noise, which might exceeds work safety requirements[3-5].

Today in an increasingly heterogeneous society educators find themselves regularly acting between the conflicting priorities of their standards and the family's socio cultural believes. Additionally documentation and administrative tasks accumulate increasingly in recent years [6]. The spatial and material conditions and the division of labour within the organization, the possibilities of participation may also play a role in the degree of perceived stress. An intensive focus on recipients and clients as typical for educational profession can lead to high workload, if organizational framework impedes relationship building [7]. Educators complain about: size of the group [8], unfavourable respectively long working hours and difficulties in contact with parents. Sixty per cent of educators in a survey judged shortage of staff to be a strain [3]. Half of them perceive time pressure and multitasking as stressful - especially managers of day care centres [5,9]. Ninety-two per cent of respondents report on to many tasks whereas they do not feel enough equipped for it [10]. One in five complain about interruptions [11] which could lead to deterioration of educational tasks.

Communication and participation are resources of the work in kindergartens [8]. Studies showed a close connection between perceived occupational competence and strains resulting from conflicts with parents [12]. Furthermore possibilities of a personal work style and work with children are sources of energy and motivation.

The health situation of educators differs from average values of the labour force of Germany. Educators possess more sick days in overall comparison to other staff in public service. Forty per cent of these sick days are caused by musculoskeletal disorders [2]. Mental and psycho-somatic disorders also appear more frequently than in the average population [13]. The most frequent complaints include: shoulder and neck pain, sacroiliac or back pain, musing, languor, and uneasiness [11]. As a consequence of job conditions educators are also susceptible to develop job burnout [3,5]. These occupational demands may as well have an influence on educators' work ability.

### Work ability of educators

Ilmarinen and Tempel define work ability as the sum of factors enabling an employed person in a certain situation to manage his or her working demands successfully [14]. That means work ability is a dynamic process following an interaction of working conditions, individual characteristics and society [15,16]. The preservation of work ability is not only a consequence of health and functional capacity, but also a result of work demand and organization [17]. Research have shown, that for educators in general aspects of work like lack of support, bureaucracy, time pressure and workload have negative effects on health [18-20].

The effects of work-related and personal risks and resources on educators' work ability in day care centres have not been well researched. Moreover it is unclear if the ownership type of the day care centres have an influence on job satisfaction and work ability of employees. Available studies on workload and strain in this profession were based on questionnaires and statements of educators. Objective data is not available [5,6]. Nevertheless a probable divergence can be expected between findings from of objective and subjective observation.

### Computer based job task analysis method

Job task analyses are used to quantify work flow. Aim is to document demands of a specific job. The analysis can form a base for future job improvements [21].

This approach is defined by examining the tasks and sequences of tasks necessary to perform the job within a defined period of time. Usually a stopwatch, pencil and protocol sheet was used [22]. But this method is time consuming and imprecise [23]. These problems can be reduced by a computer-based recording methodology. It enables the collection of different tasks truthfully to the nearest second [24-29]. Mache et al. recently developed a job task analysis instrument for monitoring physicians' job tasks in different medical settings. Analyses revealed methodology's validity and reliability for evaluating physicians' working routines as well as organizational context factors [26]. This job task analysis instrument can be extended to other professions [26] and will be used for the present study. The software and the task categories will be adapted for the present research question. This adjustment includes:

• Generation of a set of categories for educators' task analysis

• Definition of body postures assumed during work time

Many educators complain about physical strain caused by their work [8,10,30]. Due to the fact that it is unknown how time an educator has to spent in different body positions, recording of different body positions and movements will be part of the intended monitoring.

### Aims and objectives

The main objective of this investigation is to collect precise time data of educators' work task.

With the help of the intended study accurate job task profiles ought to be defined with a view on different kind of ownership. Multi-level diagnostic measurement is used to identify possible sources of stress i.e. educators' work load and occupational demands. It is a long-term goal to develop approaches for prevention based on the results of the study. Therefore an identification of ranges of stress is needful.

To achieve this goal bidirectional assessment is applied. It permits evaluation of workload objectively independent of educators' point of view. In addition the subjective perception of working conditions will be captured. This makes it possible to assess aspects of work and personal as well as organisational resources which may have an influence on educators' work ability and job satisfaction.

Within the framework of the investigation a set of questions will be answered.

1. What kind of occupational demands exist for educators in day care centres?

2. Does the type of ownership have an influence on educators' working conditions (e.g. occupational demands, state of health) and job satisfaction?

3. What kinds of resources exist in this profession?

4. Are there differences in workload with regard to different types of ownership?

5. Do different kinds of organisation models have an effect on work flow?

6. What factors have an impact on educators' job satisfaction?

7. What kind of occupational resources have an influence on work ability of employees?

## Method

### Sample and Recruitment

**Figure 1:**
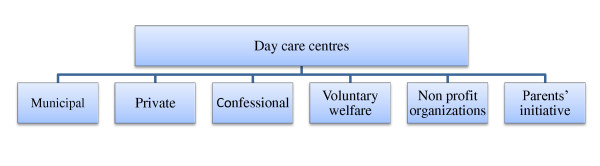
**Types of ownership of German day care centres**.

Educators caring for children of different age groups (0-6 years) will be included in the study. Furthermore, they will be recruited from day care centres of different ownership types (private, municipal, confessional, voluntary welfare, non profit organizations, and parents' initiative) (Fig. 1).

After contacting management of day care centres by the research group, educators are in invited to participate in the study on a voluntary basis. The comparative samples are composed of educators working at day care centres of different ownership. In so doing a satisfying comparability of the groups will be achieved.

### Instruments

#### Objective task-analysis

Objective task analysis is based on data of educators' work in day care centres. These data forms the basis for load analyses. In so doing a differentiation can be made between subjects with very stressful or less stressful jobs. This discrimination can be performed most precisely via objective task analyses.

**Figure 2:**
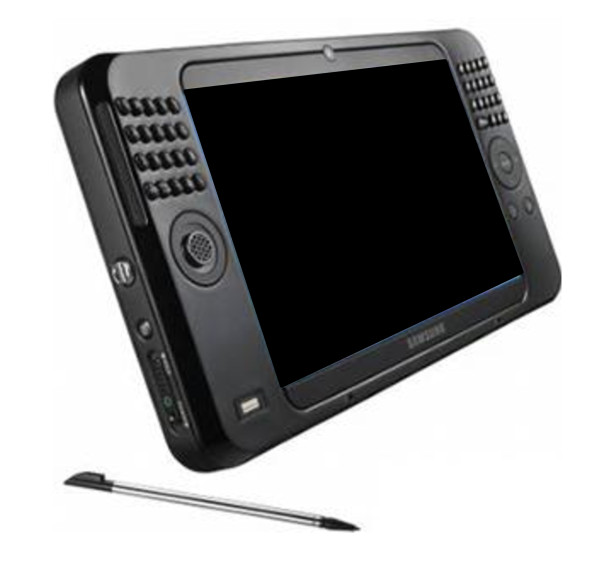
**Ultra Mobile PC**.

With the aid of the developed activity acquisition program sequence of work can be recorded by mobile computer-based collection equipment (Ultra Mobile PC) (Fig.2).

This activity acquisition program is based on tasks what educators perform in their daily work routine. First step to obtain this taxonomy is a literature review on job tasks. In a second step interviews with conversant specialists in these areas have to be conducted. The taxonomy makes objective work flow documentation possible. Additionally physical strain will be registered by recording body posture employees assume during their work time.

The Ultra Mobile PC can operated singlehanded and permits the exact time registration by touching the screen. After monitoring, data will be analysed statistically and graphically: the number of individual occurrences of each task, mean duration of each occurrence, the total time (in seconds) spent on each task, task category, and the time expenditure for all tasks will be counted.

Significant differences will be detected with a sample of at least 15 educators of day care centres of different type of ownership. They will be observed during three workdays. In doing so every weekday should be evenly distributed so much that a comparison of educational tasks is achievable for all weekdays.

Subsequently the collected data will be interpreted and used as reference values for educators work.

During monitoring the following aspects should be documented:

• The kind of work activity

• Duration and frequency of the tasks

• Body postures assumed during work time

• Work environment (noise level, lighting, temperature)

• Interruptions (frequency, duration, type)

• Rest periods (time, duration, location)

#### Job demands and work stress - a questionnaire study

The study design enables bidirectional assessment of objective and subjective perception of the work situation of educators. In so doing not only job demands will be measured but also personal resources which might have an influence on work ability, job satisfaction and the perception of stress. Moreover only job holders themselves can evaluate their working conditions and workload.

These job related factors at work will be measured with the German version of the Copenhagen psychosocial questionnaire (COPSOQ) [31]. This instrument covers multiple aspects of work, for example:

• Demands (e.g. emotional demands, work - privacy conflict)

• Influence and development (e.g. meaning of work, workplace commitment)

• Interpersonal relations and leadership (e.g. feedback, social relations)

• Outcomes (e.g. job satisfaction, burnout)

Additionally the Work Ability Index (WAI) [32] will be used to assess how long educators are able to work and to what extent this ability depends on the work content and job demands.

The self-administered questionnaire will be provided to educators as paper-and-pencil form. Participants will be asked to fill in and post it in a box positioned in their workplace within two weeks.

### Statistical data analysis

Subject to the hypotheses different kinds of statistical analyses will be performed:

Basic statistics and ANOVAs (analysis of variance) will be calculated to analyse differences in work conditions as well as in job satisfaction and work ability between different ownership types.

Hierarchical regression analysis will be applied to analyse the degree to which work ability and job satisfaction of educators can be explicated by job demands and resources. In order to assess differences in the effects of job demands and resources between the day care centres, discrete analyses will be carried out for each type of day care centres ownership.

All tests will be applied two-tailed. P-values < .05 will be considered statistically significant throughout. Values will be given as mean and standard deviation. Data analysis will be performed using SPSS^® ^software package for social sciences; Version 16.0.

## Discussion

As far as we know no computer-based real time studies do exist on work activity in day care centres in order to objectively evaluate an educator's work-related stress. Main objective is to identify sources of stress and to develop approaches for prevention based on the results of the study. It is a long-term goal to create a health-promoting work environment for educators. This may result an increased job satisfaction and work ability of educators. Altogether this study can substantially contribute to an overall improvement of child care in Germany.

## Competing interests

The authors declare that they have no competing interests.

## Authors' contributions

BK, SM and DAG conceived and designed the study. BK wrote the manuscript. BK, AN, MS, DQ, DAG, and SM contributed substantially to its final version. All authors read and approved the final manuscript.

## References

[B1] Seibt R Khan A Netzwerk für gesunde Beschäftigte in Kindertagesstätten 2005 Dresden: Institut Poliklinik für Arbeits- und Sozialmedizin

[B2] Schad M Erziehung (k)ein Kinderspiel. Gefährdungen und Belastungen des pädagogischen Personals in Kindertagesstätten Band 7 der Schriftenreihe der Unfallkasse Hessen 2002 Frankfurt am Main: Unfallkasse Hessen

[B3] BKK-BV Gesundheitsbericht 1993 BKK Stadt Kassel 1993 Essen: Bundesverband der Betriebskrankenkassen. Abteilung Gesundheit - Referat Gesundheitsberichterstattung

[B4] Buch M Frieling E Badura B, Litsch M, Vetter C Ableitung und Evaluation von Arbeitsgestaltungsmaßnahmen bei ErzieherInnen in Kindertagesstätten Fehlzeiten-Report 2001 Zahlen, Daten, Analysen aus allen Branchen der Wirtschaft Gesundheitsmanagement im öffentlichen Sektor 2001 Berlin: Springer 103 118

[B5] Rudow B Belastungen und der Arbeits- und Gesundheitsschutz bei Erzieherinnen. Langfassung des Projektberichtes 2004 Mannheim & Mühlhausen/Thür.: Institut für Gesundheit und Organisation (IGO)

[B6] Gewerkschaft Erziehung und Wissenschaft Wie gehts im Job? KiTa-Studie der GEW 2007 Frankfurt am Main: Gewerkschaft Erziehung und Wissenschaft - Hauptvorstand

[B7] Gewerkschaft Erziehung und Wissenschaft Der Arbeitsschutz für Erzieherinnen in Kindertagesstätten 2005 Stuttgart Gewerkschaft Erziehung und Wissenschaft - Baden-Württemberg

[B8] Botzet M Frank H Arbeit und Gesundheit von Mitarbeiterinnen in Kindertageseinrichtungen. Regionalfallstudie in saarländischen Kindertageseinrichtungen 1998 Saarbrücken: Landesarbeitsgemeinschaft für Gesundheitsförderung e.V.

[B9] Dippelhofer-Stiem B Kahle I Empirische Analysen zur pädagogischen Arbeit im Kindergarten Zeitschrift für Frauenforschung 1994 12 111 122

[B10] Rudow B Arbeitsbedingungen für Erzieher/innen. Hohe psychische Belastungen Bildung und Wissenschaft 2004 6 13

[B11] Berger J Niemann D Nolting H-D Schiffhorst G Genz HO Kordt M Stress bei Erzieher/innen. Ergebnisse einer BGW-DAK-Studie über den Zusammenhang von Arbeitsbedingungen und Stressbelastung in ausgewählten Berufen 2004 Hamburg: BGW; DAK

[B12] Dippelhofer-Stiem B Kahle I Die Erzieherin im evangelischen Kindergarten 1995 Bielefeld: Kleine

[B13] Nolting H-D Berger J Niemann D Schiffhorst G Genz HO Kordt M Stress bei Kindergärtner/innen 2000 Hamburg: BGW; DAK

[B14] Ilmarinen J Tempel J Arbeitsfähigkeit 2010. Was können wir tun, damit Sie gesund bleiben (Work ability 2010. What can we do so that you remain well)? 2002 Hamburg: VSA

[B15] Ilmarinen J Tuomi K Eskelinen L Nygard CH Huuhtanen P Klockars M Background and objectives of the Finnish research project on aging workers in municipal occupations Scand J Work Environ Health 1991 17 Suppl 1 7 11 1792532

[B16] Ilmarinen J Costa G Aging of the working population in the European Union Med Lav 2000 91 279 295 11098592

[B17] Ilmarinen JE Aging workers Occup Environ Med 2001 58 546 552 10.1136/oem.58.8.546 11452053 PMC1740170

[B18] Sarmiento TP Laschinger HK Iwasiw C Nurse educators' workplace empower-ment, burnout, and job satisfaction: testing Kanter's theory J Adv Nurs 2004 46 134 143 10.1111/j.1365-2648.2003.02973.x 15056326

[B19] Burke RJ Greenglass E Work stress, role conflict, social support, and psycho-logical burnout among teachers Psychol Rep 1993 73 371 380 8234588 10.2466/pr0.1993.73.2.371

[B20] Fong CM A longitudinal study of the relationships between overload, social sup-port, and burnout among nursing educators J Nurs Educ 1993 32 24 29 8380202 10.3928/0148-4834-19930101-07

[B21] Cascio W Aguinis H Applied Psychology in Human Resource Management 2005 6 Upper Saddle River: Prentice Hall

[B22] Fine SA Cronshaw SF Functional job analysis: A foundation for human re-sources management 1999 Mahwah, NJ: Erlbaum

[B23] De Leeuw E Nicholls W Technological Innovations in Data Collection: Accep-tance, Data Quality and Costs Sociological Research Online 1996 1

[B24] Mache S Bernburg M Scutaru C Quarcoo D Welte T Klapp BF Groneberg DA An observational real-time study to analyze junior physicians' working hours in the field of gastroenterology Z Gastroenterol 2009 47 814 818 10.1055/s-0028-1109175 19750428

[B25] Mache S Jankowiak N Scutaru C Groneberg DA Always out of breath? An analysis of a doctor's tasks in pneumology Pneumologie 2009 63 369 373 10.1055/s-0029-1214798 19591082

[B26] Mache S Scutaru C Vitzthum K Gerber A Quarcoo D Welte T Bauer TT Spallek M Seidler A Nienhaus A Development and evaluation of a computer-based medical work assessment programme J Occup Med Toxicol 2008 3 35 10.1186/1745-6673-3-35 19094213 PMC2628342

[B27] Mache S Scutaru C Vitzthum K Quarcoo D Schoffel N Welte T Klapp BF Grone-berg DA Does type of hospital ownership influence physicians' daily work sched-ules? An observational real-time study in German hospital departments Hum Resour Health 2009 7 41 10.1186/1478-4491-7-41 19473487 PMC2692979

[B28] Mache S Kelm R Bauer H Nienhaus A Klapp BF Groneberg DA General and visceral surgery practice in German hospitals: a real-time work analysis on surgeons' work flow Langenbecks Arch Surg 2010 395 81 87 10.1007/s00423-009-0541-5 19618203

[B29] Mache S Vitzthum K Kusma B Nienhaus A Klapp BF Groneberg DA Pediatricians' working conditions in German hospitals: a real-time task analy-sis Eur J Pediatr 2010 169 551 555 10.1007/s00431-009-1065-2 19774393

[B30] Buch M Frieling E Belastungs- und Beanspruchungsoptimierung in Kindertagesstätten 2001 Kassel: Institut für Arbeitswissenschaft

[B31] Nübling M Stossel U Hasselhorn HM Michaelis M Hofmann F Measuring psychological stress and strain at work - Evaluation of the COPSOQ Questionnaire in Germany Psychosoc Med 2006 3 Doc05 PMC2736502 19742072

[B32] Tuomi K Ilmarinen J Jahkola A Katajarinne L Tulkki A Arbeitsbewältigungsindex - Work Ability Index 2001 Bremerhaven: Wirtschaftsverlag NW Verlag für neue Wissenschaft

